# Metabotropic glutamate receptor 5-related autoimmune encephalitis with reversible splenial lesion syndrome following SARS-CoV-2 vaccination

**DOI:** 10.1097/MD.0000000000032971

**Published:** 2023-02-17

**Authors:** Yu Zhang, Baorong Lian, Shiwei Yang, Xuming Huang, Yanxia Zhou, Liming Cao

**Affiliations:** a Department of Orthopaedics, Zhejiang Hospital, Hangzhou, China; b Clinical College of the Shenzhen Second People’s Hospital, Anhui Medical University, Shenzhen, China; c Shantou University Medical College, Shantou University, Shantou, China; d Teaching office, The First Affiliated Hospital of Shenzhen University, Shenzhen, China; e Department of Internal Medicine, Shenzhen Shiyan People’s Hospital, Shenzhen, China; f Department of Neurology, The First Affiliated Hospital of Shenzhen University, Shenzhen, China; g Hunan Key Laboratory of the Research and Development of Novel Pharmaceutical Preparations, Changsha Medical University, Changsha, China.

**Keywords:** acute respiratory syndrome coronavirus 2, autoimmune encephalitis, case report, metabotropic glutamate receptor 5, reversible splenial lesion syndrome

## Abstract

Metabotropic glutamate receptor 5 (mGluR5)-related autoimmune encephalitis (AE) has been rarely reported; however, there are no reports on mGluR5-related AE with reversible splenial lesion syndrome following vaccination against severe acute respiratory syndrome coronavirus 2 (SARS-CoV-2).

**Patient concerns:**

A 29-year-old man was admitted with a history of headache and fever for 9 days and 6 days, respectively.

**Diagnosis:**

He was initially diagnosed with an intracranial infection, however the final diagnosis was corrected as anti-mGluR5-related AE with reversible splenial lesion syndrome.

**Interventions:**

He had received an inactivated SARS-CoV-2 vaccine 3 weeks prior to the examination and was initially diagnosed with an intracranial infection. Physical examination revealed bilateral horizontal nystagmus, ataxia, and neck rigidity. Antiinfective therapy was minimally helpful. An analysis of the cerebrospinal fluid did not reveal pathogens for sequencing. Magnetic resonance imaging displayed abnormal signals in the splenium of the corpus callosum.

**Outcomes:**

We identified mGluR5 antibodies in the cerebrospinal fluid and serum. Subsequently, intravenous methylprednisolone pulse and gamma-globulin pulse therapies were administered, which substantially improved the symptoms. Follow-up did not reveal abnormal neurological symptoms, and the lesion in the corpus callosum had resolved.

**Lessons:**

AE with mGluR5 antibodies could arise from SARS-CoV-2 vaccination, which warrants the awareness of healthcare workers. Reversible splenial lesion syndrome may accompany mGluR5-related AE and mimic intracranial infection. Thus, early treatment can prevent serious residual signs and symptoms.

## 1. Introduction

Metabotropic glutamate receptor 5 (mGluR5)-related autoimmune encephalitis (AE) has been rarely reported; however, there are no reports on mGluR5-related AE with reversible splenial lesion syndrome (RESLES) following vaccination against severe acute respiratory syndrome coronavirus 2 (SARS-CoV-2). The World Health Organization has declared SARS-CoV-2 infection a worldwide pandemic. Prevention with vaccines may be a better option than treatment following infection. Thus, the SARS-CoV-2 vaccine is being administered on a global scale. Researchers have reported a spectrum of neurological complications following SARS-CoV-2 vaccination, such as acute myelitis, acute disseminated encephalomyelitis,^[1]^ and acute demyelinating polyneuropathy.^[2]^ Symptom onset occurs 1 to 3 weeks following the final vaccine dose.

mGluR5-related AE is a rare autoimmune-mediated central nervous system disease. The major clinical manifestations include psychiatric symptoms such as behavioral changes, memory and cognitive dysfunction, seizures, and movement and consciousness disorder.^[3,4]^ The most frequent psychiatric manifestations are behavioral or personality/mood changes, ranging from irritability or agitation to severe anxiety, depression, and full-blown psychosis with abnormal thought processes and hallucinations.^[4]^ RESLES is a spectrum of disorders, which is radiologically characterized by reversible lesions present in the splenium of the corpus callosum.^[5]^ RESLES can be accompanied by seizures, encephalopathy, headache, and ataxia. AE is a rare condition associated with RESLES.^[6–9]^ Currently, there are no reports on the occurrence of mGluR5-related AE with RESLES after SARS-CoV-2 vaccination. Herein, we present a similar case and review the literature to improve the diagnosis and treatment.

## 2. Case description

A 29-year-old man was admitted to a local hospital in June 2021. For 9 days prior to admission, he suffered from headaches, dizziness, muscle soreness, hyperthermia (40°C), weakness, and decreased appetite after being in the rain. He had received an inactivated SARS-CoV-2 vaccine (Vero Cells, Beijing Institute of Biological Products Co., Ltd., Beijing, China) 3 weeks prior to admission and did not have a history of major trauma, toxic exposure, smoking, alcoholism, drug abuse, or hereditary disease. The patient was diagnosed with an intracranial infection based on computed tomography scan results and symptomology. He was treated with antibiotics (ceftriaxone) and antiviral therapy (ganciclovir) for 5 days. While the headache was slightly relieved, other symptoms did not improve. Subsequently, he was transferred to our hospital.

**Figure 1.**
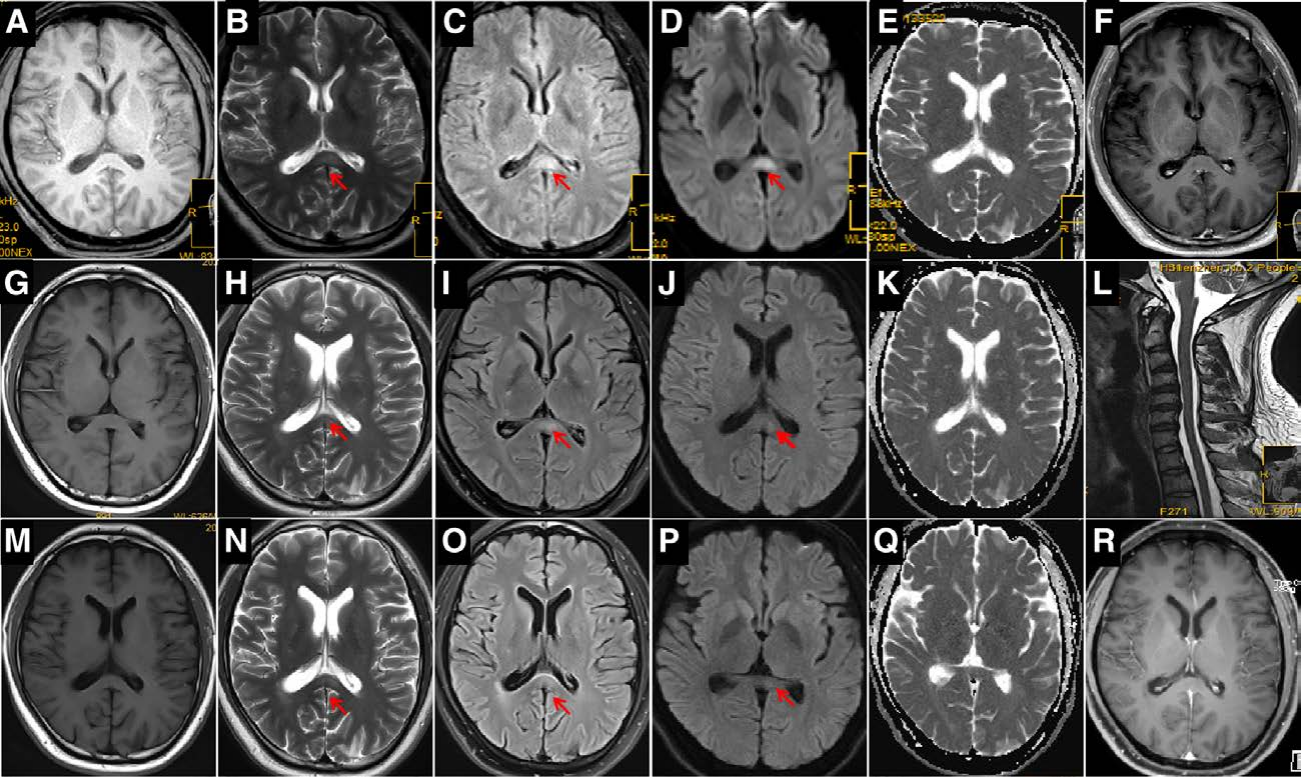
Changes in magnetic resonance imaging (MRI) findings of the patient on day 3. (a) T1-weighted imaging (WI); (b) T2-WI; (c) Fluid-attenuated inversion recovery (FLAIR); (d) Diffusion-weighted imaging (DWI); (e) Apparent diffusion coefficient (ADC); and (f) Enhanced T1-WI. (b–d), MRI displaying abnormal signals in the splenium of the corpus callosum (arrows). (g–i), MRI on day 7. (g) T1-WI; (h) T2-WI; (i) FLAIR; (j) DWI; (k) ADC; (l) Cervical spine T2-WI; (h–j), Abnormal MRI signals in the splenium of the corpus callosum display substantial improvement (arrows). (m–r), Repeat MRI at 3 weeks postdischarge. (m) T1-WI; (n) T2-WI; (o) FLAIR; (p) DWI; (q) ADC; (r) Enhanced T1-WI; (n–p), Dissipation of abnormal MRI signals in the corpus callosum (arrows). MRI = magnetic resonance imaging.

Neurological examination on admission revealed horizontal nystagmus in both eyes, positive Romberg sign, neck rigidity, and an inability to complete the finger-to-nose and heel-knee-tibia tests. There were no other obvious abnormalities. The Mini-Mental State Examination Scale score was 28/30, and the Self-Rating Anxiety Scale score was 65/100, indicating moderate anxiety. Table 1 summarizes the laboratory findings. Routine chest computed tomography revealed scattered linear lesions in both lungs. Routine abdominal ultrasonography revealed a mildly fatty liver. Moreover, we observed unusually slow electroencephalographic waves, indicating an abnormal result. Brainstem auditory evoked potential tests indicated that the brainstem auditory pathways were slightly damaged bilaterally. Brain magnetic resonance imaging (MRI) on day 3 displayed abnormal signals in the splenium of the corpus callosum (Fig. 1a–f).

**Table 1:** Laboratory findings.

	Result	Reference range	
At the local hospital			
WBC count in blood, ×10^9^/L	11.52	3.5–9.5	Increased
Neutrophil, ×10^9^/L	7.46	1.8–6.1	Increased
Leukocytes in CSF, ×10^6^/L	297	0–8	Increased
Protein in CSF, mg/L	1460	150–450	Increased
Glucose level in CSF, mmol/L	3.44	2.5–4.5	Normal
At our hospital			
Monocyte count, ×10^9^/L	0.72	0.1–0.6	Increased
Erythrocyte count, ×10^12^/L	6.12	4.3–5.8	Increased
Eosinophil count, ×10^9^/L	0.63	0.02–0.52	Increased
Thyrocalcitonin level, ng/mL	0.098	<0.094	Increased
Serum creatinine level, μmol/L	77.1	57–97	Normal
Alanine aminotransferase, U/L	54.0	9–50	Increased
Serum simplex virus IgG, s/co	>30	<1.0	Increased
Serum simplex virus IgM, s/co	0.57	<1.0	Normal
Serum cytomegalovirus IgG, U/mL	61.2	<14.0	Increased
Serum cytomegalovirus IgM, U/mL	<5.0	<22.0	Normal
Serum rubella virus IgG, IU/mL	44.2	<10.0	Increased
Serum rubella virus IgM, AU/mL	<10.0	<25.0	Normal
EBV capsid antigen IgG, U/mL	742.0	0–20	Increased
EBV nuclear antigen IgG, U/mL	>600.0	0–20	Increased
Serum ferritin, ng/mL	1398.0	21.81–274.66	Increased
Alpha-fetoprotein, ng/mL	3.7	<8.78	Normal
Serum carcinoembryonic antigen, ng/mL	1.88	<5.0	Normal
Serum prostate specific antigen, ng/mL	0.05	<0.93	Normal
Serum carbohydrate antigen 199, U/mL	15.6	<37	Normal
Serum carbohydrate antigen 153, U/mL	22.4	<31.3	Normal
WBC count in CSF on day 2, ×10^6^/L	222	0–8	Increased
Protein in CSF on day 2, mg/L	3305.2	150–450	Increased
Glucose level in CSF on day 2, mmol/L	2.08	2.5–4.5	Normal
Protein in CSF on day 8, mg/L	631.9	150–450	Increased
Protein in CSF on day 14, mg/L	593.2	150–450	Increased
WBC count in CSF on day 14, ×10^6^/L	49	0–8	Increased
Six months after discharge			
Serum carcinoembryonic antigen, ng/mL	0.64	<5.0	Normal
Serum prostate specific antigen, ng/mL	0.18	<0.93	Normal
Serum carbohydrate antigen 199, U/mL	6.92	<37	Normal
Serum carbohydrate antigen 153, U/mL	23.5	<31.3	Normal
Serum tumor associated antigen 125, U/mL	5.68	<35	Normal

CSF = cerebrospinal fluid, EBV = Epstein–Barr virus, IgG = immunoglobulin G, IgM = immunoglobulin M, WBC = white blood cell.

The patient was still under consideration for intracranial infection and continued the previously prescribed antiinfective treatment (intravenous [IV] acyclovir 1.5 g/day, IV ceftriaxone 2 g/day) and treatment to reduce the intracranial pressure (IV mannitol 75 g/day); however, the effects were not immediately obvious. Moreover, he developed psychomotor agitation, intermittent confusion, intractable hiccups, and worsening headache. He was subsequently administered IV dexamethasone (5 mg/day) on day 3 at our facility, following which his headache partly improved. Smear and culture findings of cerebrospinal fluid (CSF) were negative. CSF testing identified herpesvirus-4 (1 sequence number; reference range: <8 sequence numbers), but no other pathogens were detected by high-throughput genome sequencing. The lack of improvement in the symptoms and negative laboratory test results did not support the diagnosis of intracranial infection.

We performed AE antibody testing via a combination of cell- and tissue-based assays (Guangdong Oumeng V medical laboratory). AE antibodies, including antigamma- aminobutyric acid B receptor, antiglutamate receptor (such as NMDA, AMPA1, and AMPA 2), antileucine-rich glioma inactivated protein 1, anticontact protein-associated protein 2, and antiglial fibrillary acidic protein antibodies were negative in the CSF and serum; however, antimGluR5 antibodies were detected (titer 1:10; Fig. 2). antiaquaporin-4, antimyelin basic protein, and antimyelin oligodendrocyte glycoprotein were negative in the blood. We diagnosed antimGluR5-related AE. Subsequently, the patient received methylprednisolone pulse therapy (500 mg/day for 5 days, 250 mg/day for 5 days, 120 mg/day for 5 days, and 60 mg/day for 5 days), IV gamma-globulin (32,500 mg/day for 5 days) (400 mg/kg), a potassium supplement, antacids (stomach ulcer prevention), and symptomatic management during hospitalization. We observed substantial improvement in symptoms and lesions in a repeat MRI on day 7 (Fig. 1g–k) and improvement in the repeat CSF test results (Table 1). MRI of the cervical (Fig. 1l) and thoracic spine did not reveal obvious abnormalities.

**Figure 2.**
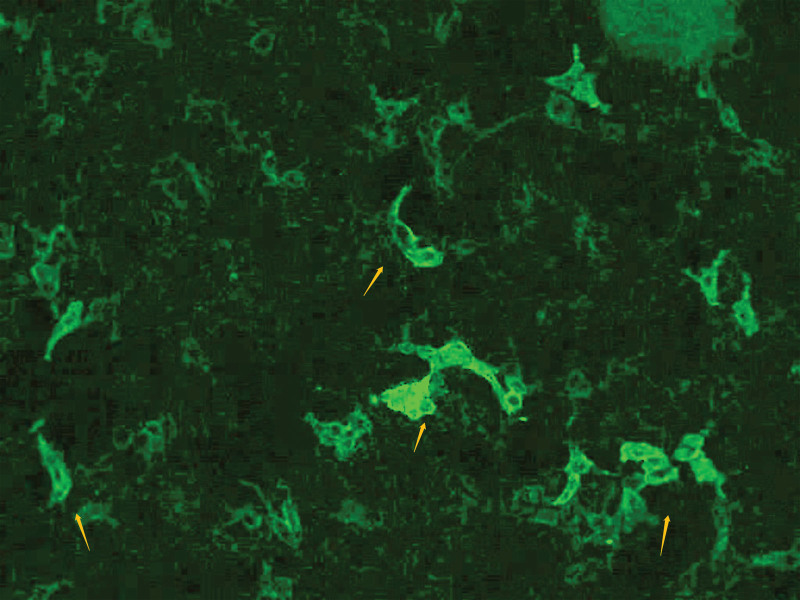
In vitro visualization of autoantibody binding using an in vitro cell based assay system. Transfected human embryonic kidney cells with metabotropic glutamate receptor 5 react with the antibodies in the patient serum and show granular fluorescence (yellow arrows, 1: 10) in a cell-based assay on admission.

The patient was discharged on day 17 and only reported fatigue and abdominal distension. He received prednisone (50 mg/day) for 2 months post discharge, following progressive dosage tapering. Repeat MRI 3 weeks after discharge demonstrated a minimal lesion in the corpus callosum (Fig. 1m–r). Thus, we considered an additional diagnosis of RESLES. At the 3-month follow-up after discharge, the patient did not demonstrate abnormal neurological symptoms; however, the mGluR5 antibody titer remained at 1:10. We performed further follow-up and repeated mGluR5 antibody testing of the blood and CSF, which were negative 6 months post discharge. Repeat MRI of the head, cervical spine, and thoracic spine showed no evidence of lymphoma, and the patient reported no discomfort. The patient was satisfied with his treatment and recovery.

## 3. Discussion and conclusions

mGluR5-related AE may accompany RESLES, which is possible after SARS-CoV-2 vaccination. Thus, awareness of healthcare workers is warranted to consider testing for mGluR5-related AE in patients presenting with intracranial infection symptoms. However, it has not been previously reported. We searched the PubMed database for the following medical subject headings: vaccines, corpus callosum, syndrome, encephalitis, receptors, metabotropic glutamate, and autoimmune diseases in different combinations; however, there were no cases similar to the present case.

There was a correlation between the time of vaccination and the onset of mGluR5-related AE in our patient. There have been reports of a spectrum of neurological complications following SARS-CoV-2 vaccination; they often occur within 1 to 3 weeks following vaccine administration.^[1,2,10]^ There are currently no other plausible causes for mGluR5-related AE in this patient; therefore, we strongly speculate that this developed following vaccine administration. It is not yet possible to draw conclusions about any significant association between SARS-CoV-2 vaccination and mGluR5-related AE. Cases similar to ours and population cohorts should be scrutinized to ensure the constant evaluation of such risks.

The mGluR5 antibody titer in our patient was low during hospitalization. Further antibody testing was performed, no mGluR5 antibody was detected in the blood and CSF at 6 months post discharge. Therefore, we consider that the low mGluR5 titer was not an epiphenomenon in the setting of an inflammatory central nervous system condition in this patient. With a further in-depth study of mGluR5-related AE patients in the future, the relationship between the low titer antibodies and the onset of mGluR5 can be clarified.

The presence of antimGluR5 antibodies can mimic intracranial infection symptoms, such as high fever, severe headache, neck rigidity, an increase in white blood cell count, and the presence of proteins in the CSF. Early treatment can result in better outcomes. Based on his presentation, our patient was rapidly diagnosed and was administered immunotherapy, thereby shortening the duration and progression of symptoms. Clinicians may misdiagnose patients with mild psychiatric symptoms or with conditions that mimic intracranial infection. This necessitates a timely and thorough examination and diagnosis to prevent complex pathology. The treatment of mGluR5-related AE requires immunotherapy. Besides neurological relapses, mGluR5-related AE patients should be closely and regularly followed up to screen for tumors, particularly Hodgkin lymphoma. mGluR5-related AE is a rare autoimmune-mediated pathology of the central nervous system and shows a good response to immunotherapy. Hodgkin lymphoma increases the risk of mGluR5-related AE,^[3]^ which can occur with no evident tumor.^[4]^ mGluR5 is primarily expressed in the hippocampus and amygdala, where it mediates numerous functions in both the peripheral and central nervous systems, including pain perception, anxiety, behavioral learning, and memory.^[11]^ antimGluR5 antibodies are associated with complex neuropsychiatric syndromes. mGluR5-related AE has a high degree of clinical heterogeneity, as most patients experience a prodromal viral-like phase, similar to our patient, followed by the development of psychotic symptoms, behavioral changes, memory and cognitive dysfunction, seizures, and movement/consciousness disorders.^[4]^

mGluR5-related AE may present with symptoms similar to RESLES, a spectrum of disorders radiologically characterized by reversibly present lesions that involve the splenium of the corpus callosum.^[5]^ These lesions can cause seizures, encephalopathy, headache, and ataxia. mGluR5-related AE and RESLES comorbidity have not been previously reported. Other types of AE associated with RESLES have been reported – 1 case each of antiN-methyl-D-aspartate receptor encephalitis, antivoltage-gated potassium channel encephalitis, antiYo encephalitis,^[6–8]^ and glial fibrillary acidic protein astrocytopathy.^[9]^ RESLES is primarily associated with antiepileptic medication withdrawal, seizures, and systemic or central nervous system infections.^[5]^ The exact etiology and pathogenesis of RESLES are not determined yet. Glutamate is principally an excitatory neurotransmitter that binds to the glutamate receptor and is responsible for approximately half of the cell-to-cell synaptic signaling in the central nervous system.^[12]^ The neurotoxicity of excitatory amino acid-induced cellular edema in the splenium of the corpus callosum may play a key role in RESLES. Thus, mGluR5-related AE may be added to the list of potential causes of RESLES. The patient had a high fever with symptoms of cold before admission, and an upper respiratory tract infection could have triggered RESLES. Other causes of RESLES were excluded during diagnosis. We conclude that mGluR5-related AE may accompany RESLES and is possible following SARS-CoV-2 vaccination.

The limitation of this study is that the relationship between low titer antibody and the onset of mGluR5-related AE remains unclear and requires further investigation. In addition, the relationship between RESLES and mGluR5-related AE needs to be further confirmed in future studies.

In conclusion, mGluR5-related AE is a rare autoimmune-mediated central nervous system disease but has not been reported following SARS-CoV-2 vaccination. mGluR5-related AE may accompany RESLES and can mimic intracranial infection. A prompt and accurate diagnosis is required to initiate early treatment and prevent serious residual symptoms. Further study is warranted to evaluate the incidence and pathogenesis of SARS-CoV-2 vaccine-mediated antimGluR5-related AE.

## Author contributions

**Conceptualization:** Yu Zhang, Baorong Lian, Shiwei Yang, Xuming Huang, Liming Cao.

**Formal analysis:** Yu Zhang, Baorong Lian, Shiwei Yang, Xuming Huang, Yanxia Zhou, Liming Cao.

**Funding acquisition:** Shiwei Yang, Liming Cao.

**Visualization:** Yanxia Zhou.

**Writing – original draft:** Yu Zhang, Baorong Lian, Shiwei Yang, Xuming Huang.

**Writing – review & editing:** Liming Cao.
